# Trauma and mental disorder: multi-perspective depictions in *Top Boy*

**DOI:** 10.3389/fpsyt.2024.1343435

**Published:** 2024-02-13

**Authors:** Wesley Quadros, Adegboyega Ogunwale, Akeem Sule

**Affiliations:** ^1^ East and North Hertfordshire NHS Trust, Stevenage, United Kingdom; ^2^ Association of Black Psychiatrists (ABP), Derby, United Kingdom; ^3^ Neuropsychiatric Hospital, Aro, Abeokuta, Nigeria; ^4^ Institute of Psychiatry, Psychology and Neuroscience, King’s College, London, United Kingdom; ^5^ Wolfson College, University of Cambridge, Cambridge, United Kingdom; ^6^ Essex Partnership University NHS Foundation Trust, Essex, United Kingdom; ^7^ Department of Psychiatry, Addenbrookes Hospital, University of Cambridge, Cambridge, United Kingdom

**Keywords:** trauma, mental disorder, media depiction, substance use, gang involvement, syndemic

## Abstract

Psychiatry has often had an uneasy relationship with popular culture as depictions of mental health may be stigmatising and inaccurate. A recent critically acclaimed series, Top Boy, set in a crime-filled fictional housing estate in the London Borough of Hackney offers an informed and fairly balanced insight into broad mental health-related themes including racial trauma embodied in social inequities, the syndemic of mental disorder, substance misuse and gang-based crime as well as the psychosocial ramifications of illustrated mental health conditions. From both idiographic and nomothetic perspectives, Top Boy touches on a rich variety of structural determinants of mental health, as well as individual and environmental predisposition to mental disorder and substance misuse. The show offers an opportunity for education for both the broader society and the groups which suffer these syndemics. An understanding of how structural factors epidemiologically affect what psychiatric conditions individuals are likely to suffer, how they can be better reached by psychiatric services, and what interventions can help improve the socioeconomic factors that lead to the behaviours/paths that individuals end up is vital for public mental health policy.

## Background

Psychiatry has often had an uneasy relationship with popular culture as depictions of mental health may be stigmatising and inaccurate (1). Mental health portrayals in pop culture will likely influence the attitudes of the general populace to mental illness and its sufferers (2). Pragmatic examinations of the depiction of mental health problems in social narratives such as movies and music are therefore pivotal to the discourse of modern social psychiatry.

*Top Boy* is a critically-acclaimed show centering on the experience of a network of individuals living in a crime-filled fictional housing estate in the London Borough of Hackney, an area that has been studied already with regards to syndemics, racial differences in mental health and the impacts of violence and gang affiliation (3). The social fabric into which syndemics are woven is underlined by multiple socio-political challenges including economic deprivation, unemployment, substandard housing or homelessness, dysfunctional family life, impaired social relationships and poor healthcare access, among others (4, 5).

Hackney provides a naturalistic setting for the show due both to its demographics and the rich quantity of research that has been done in the area in relation to syndemics and mental health. Hackney is the third most densely populated local authority area across England, with 21.1 percent of individuals identifying their ethnic group as Black, Black British, or Black Caribbean or African category in 2021 (6). Despite providing more affordable housing than any other London borough, Hackney had one of the highest rates of homelessness in London as recently as 2015 (7). With regard to young people, 48 percent of children in the Borough were living below the poverty line in 2019/2020, the 4th highest among all parliamentary constituencies in the UK (8). The ability of the state to intervene to help individuals in areas like Hackney has been hampered by years of austerity which have hollowed out local government infrastructure. Local councillors estimate cuts in Hackney Council at almost half the size of its budget (9).

In the show, the ‘*Top Boy*’, Dushane Hill, runs a gang, Summerhouse, involved in London’s underground drug trade, which due to the prevalence of young members has wide-ranging effects on the mental health of the individuals on the estate. *Top Boy*’s mental health depictions are of particular relevance for analysis for primarily three reasons. It provides an in-depth coverage of a number of heterogeneous mental health issues from both idiographic and nomothetic perspectives. It does this in a way that appreciates how sociocultural and economic factors at play in an individual’s life could exacerbate their psychopathology. If we are to progress in our conceptualisation of social psychiatry and how to implement effective policy interventions for mental health and gang involvement, such understanding is invaluable (10). Secondly, *Top Boy* is undoubtedly one of the most culturally successful and far-reaching examples of the ‘black urban crime’ genre, running for multiple seasons on the UK’s leading multicultural public service broadcaster Channel 4 and then Netflix (11–13). Its depictions will arguably impact discourse and perception of mental health and gang involvement among the demographics of its viewership (14), some of whom are likely to feel dissociated from mainstream society, and these are pertinent for dissection as a tool for developing better understanding the social determinants of psychiatric illness in multi-racial societies and the relevance of possible interventions. Thirdly, to our knowledge, this is the first attempt to provide a public mental health dimension to the limited discourse on *Top Boy*. Previous reviews had focused on its media impact as well as its sociological and criminological implications (14, 15).

In this paper, we discuss *Top Boy*’s depictions of broad mental health-related themes including racial trauma embodied in social inequities, the syndemic of mental disorder, substance misuse and gang-based crime as well as the psychosocial ramifications of illustrated mental health conditions. *Top Boy* touches on a rich variety of structural determinants of mental health, as well as individual and environmental predisposition to mental disorder and substance misuse. From a public mental health perspective, these structural determinants include social, cultural, economic, political and environmental factors such as social protection, living standards, working conditions, community social supports as well as the impact of exposure to adversity across the lifespan (16).

In addition to several idiographic illustrations of these determinants (or lack thereof) in the series, this paper also addresses the specific roles of domestic abuse and other forms of trauma in creating a syndemic. Concomitant structural determinants include migration difficulties as well as the effects of urbanicity and its attendant social deprivation. The resultant social disadvantage is intricately linked to drug-related crime, violence perpetration and victimisation as well as other forms of criminality as depicted in *Top Boy*. Individual and environmental predisposition to mental illness principally relates to exposure to illicit drugs and domestic violence. The significant impact of parental substance abuse (maternal drug dependence) and mental disorder (maternal depression) is also potently illustrated using inter-individual narratives in the show. We dissect how the interplay of these factors is weaved delicately through the plots in the show, and the effects they have on the mental health of its characters.

The public mental health implication of a combination of structural dysfunctions as well as individual vulnerabilities is their significant impact on mental health promotion, illness prevention as well as treatment, recovery and rehabilitation (16, 17) as is variously depicted in many individual narratives within *Top Boy*.

The effects of maternal drug dependence on adolescent psychopathology are observed in Jason, a physically and emotionally neglected young boy whose mother is homeless and drug-dependent. With exposure to repeated instances of domestic violence in the frame of neglect and abuse as well as repeated witnessing of physical violence and intimidation against himself and his mother, he gradually develops features consistent with post-traumatic stress disorder (PTSD) or even complex PTSD (c-PTSD) (18). PTSD is a stress-related disorder which occurs following exposure to an extremely threatening stimulus such as physical violence. Complex PTSD tends to occur with more protracted exposure to such trauma whether cumulatively or as single significant events (19). Both PTSD and c-PTSD are linked to traumatic experiences including interpersonal violence, and the more violence a young person is exposed to the greater the PTSD symptoms (20, 21). This dose-dependent relationship is explored as Jason’s character grows up on screen.

Jason’s symptoms of these disorders included fear and avoidance of his “home” environment although there were no scenic illustrations of other key symptoms like re-experiencing e.g. flashbacks. There were also problems with his affective regulation (his emotion appeared frequently numb), self-concept and interpersonal relationships. A combination of these resulted in his association with gangster Sully who developed a supportive, albeit antisocial, relationship with him. Jason becomes desensitised to violence probably due to the affective dysregulation and dissociative effects of PTSD or c-PTSD (22, 23). This is illustrated in a scene where Sully is very violently enacting physical punishment on another character, Jason sits and eats in the foreground in a distant and unemotional manner. It is not unlikely that Jason’s eventual slide into opiate addiction when Sully goes to prison represented an attempt to deal with on-going PTSD or c-PTSD psychopathology. Studies have documented considerable comorbidity of PTSD with drug use (24, 25). It could also be postulated that he took to drug misuse via the social modelling route given his background of parental drug abuse and gang involvement (3, 5).

Later on, Sully watches Jason burn to death after a racially motivated attack. This intricately illustrates the nested nature of such traumatic experiences as we then go on to see Sully develop PTSD himself. He demonstrates the symptoms of re-experiencing, hypervigilance and avoidance as variously depicted in the show. He is seen re-experiencing the fire and smell of smoke following Jason’s demise. He later tries to cut himself off from his affiliations with Summerhouse and retreats into living on a boat, perhaps a means of avoiding cues related to the traumatic death of his protégé. His hyperarousal is frequently illustrated by his perpetually heightened state of threat with this leading to several instances of reactive violence on his part. He equally engaged in instrumental violence largely driven by his irrational threat perception. Exposure to violence, particularly in the context of gang membership, is strongly associated with anxiety and other disorders (3).

Through the lens of public mental health, it may be seen that Jason lacked many factors that could have been mental health promoting. According to the conceptual underpinnings of mental health promotion (16), it involves the creation of individual, social and environmental conditions that facilitate optimal psychological and psychophysiological development. Jason’s life-course exposure to trauma and substance misuse robbed him of such an enabling environment and set the stage for mental health problems in his young adulthood.

Through the story of Lauryn, the show devastatingly explores an association between social isolation, domestic violence against women and maternal depression, with tragic consequences. When Lauryn is first introduced in the show as the sister to Jaq, one of the criminal masterminds of Summerhouse, she is portrayed as an assertive and confident woman with a strong social network in her community. However, her romantic involvement with a local gangster with rivalries against Summerhouse places her perilously in the midst of gang warfare. She unwittingly reveals intelligence she overhears from Jaq about Summerhouse to her boyfriend which almost results in Sully’s murder. This discovery prompts Sully to seek revenge with the intention of killing her.

Revenge is seen as a recurring theme in the show and presents itself as a fuel for protracted gang violence (26, 27). The murderous intentions of Sully made known to Lauryn by Jaq forces her into absconding from her home in order to save her life. Now far away in Liverpool and socially isolated, she falls into a relationship with a local gangster, Curtis, who is extremely coercive and manipulative. Without the protective factor of her social network and the exacerbating effect of being intimidated and bullied by Curtis’ sister, her confident and stoic exterior is slowly eroded leaving her insecure and eventually substance- dependent. A complex interplay of themes such as social isolation, physical entrapment, economic dependence and a sense of investment in the relationship (in this case, pregnancy) is observed in her experience of domestic violence within her relationship with Curtis (28). She eventually returns under a shroud of secrecy, but is trailed by Curtis who she murders in an act of self-defence. Shortly after, she gives birth to his son and falls into an episode of postnatal depression as evidenced by persistent low mood, anhedonia, poor motivation and difficulty with maternal care.

In Lauryn, we see the synergy between her risk factors, namely social isolation, domestic abuse and stress, in the evolution of the severe postnatal depression that emerges. As she falls into the grip of worsening illness, her social and occupational functioning declines with inability to cope with her parental duties which are increasingly left to Jaq. This illustrates a well-known red flag for perinatal suicide - a new or persistent expression of incompetency in parenting or estrangement from the newborn – especially against the backdrop of significant depressive symptoms (29). As she seeks more relief from her frustrations, Lauryn’s self-medication with recreational drugs intensifies. She covertly gets her drug supply from another member of the Summerhouse gang. Jaq insists she immediately stops using drugs when she discovers this and shortly after, Lauryn ends her life from an overdose.

Lauryn’s apparent post-natal depression, substance misuse and eventual perinatal suicide all stand as poignant reminders of failure in mental health promotion, mental illness prevention and early intervention. First, her background of domestic violence and gang involvement could hardly have contributed to positive mental health. Furthermore, while Jaq becomes fixated with the idea that Lauryn’s death resulted from an accidental overdose under the influence of drugs. Lauryn’s tragic story reflects the experience of a new mother facing multiple adversities including mental ill health, substance abuse, long-term effects of domestic abuse and material deprivation. This signals a lot of the issues highlighted by the UK MBRRACE report which noted increased risk of maternal mortality in ethnic minoritised women and how ethnic inequalities are not always universally reflected in data on suicides (30). The MBRRACE data showed that Black women were four times more likely, while Asian women were close to two times more likely to die than white women. When surveyed, these women often suffered from multiple structural biases in maternity care (31) and they should be a focus group with regard to the very worrying trend in rising perinatal suicides (32). In a sense, such biases have serious implications for public mental health imperatives like early identification, assessment and intervention which are crucial in suicide prevention (33).

The devastating inter-individual effects of this suicide are seen through the lens of Jaq, whose entire worldview is shaken by it. She begins to question her gang involvement, given its role in drug distribution which provided Lauryn the access to the substance which led to her death. In a sense, her pining and impulsive behaviours following Lauryn’s death perhaps represented a grief reaction. Arguably, her intense interest in a local mother who buys drugs from Summerhouse could suggest a pining for Lauryn who had died. A visit to this woman’s house reveals gross emotional and physical child neglect and an environment indicative of material deprivation and a loss of self-care. Ridden with guilt for being responsible, in a sense, for Lauryn’s death, Jaq tries to reprise the role she had played with Lauryn and sought to assume such a relationship with the Lauryn surrogate. She insists that the drug-dependent mother stops using drugs and rather focuses on child care. From Jaq’s contemporaneous experience, a perinatal suicide does not only leave a child without a mother, but also a traumatised family who must bear the grief of losing a significant love object - their kin. Notwithstanding this, Jaq’s experience of grief was not a simplified process of seeking redemption through righting the wrongs that fuelled her guilt. In one illuminating scene, her partner confronts her around the need for Jaq to pursue an alternative career instead of one of gang involvement and drug dealing. Realistically, Jaq says selling drugs is all she has ever known. Individuals living in the midst of such a syndemic environment as depicted in *Top Boy* remain limited in their social and occupational opportunities within their intractably disadvantaged existence. As such, their life options remain difficult to disentangle from anomic alternatives such as gang affiliation and other forms of criminality.

The wider impacts of trauma and violence on the family unit are explored through the story of Ra’Nell and his mother Lisa. Lisa is a single mother living with the effects of an abusive relationship with her ex-partner and develops a debilitating depressive illness with psychotic features that results in her being sectioned in an extended psychiatric admission. The effects of parental mental disorder on child development include poor self-esteem, poor academic adjustment, depression, substance use and antisocial attitudes (34). This story explores how her absence leaves Ra’Nell isolated and vulnerable to exploitation for antisocial ends. He is groomed by his mother’s acquaintance to become a help on her in-house cannabis farm. He ultimately falls into the narcotic trade of the estate’s gang, leading to truancy, physical and emotional abuse and serious violence in which he witnesses the killing of a benefactor. An extension of Ra’Nell’s exploitation leads his close friend, Gem, to attempt a personal involvement in the illegal cannabis cultivation on the estate. The plan falls through after significant commercial investment by a drug dealer who then blackmails Gem to act as a drug mule for the gang.

This plot in the show illustrates how early involvement in the drug economy leads to the huge prevalence of drug dependence among gang members (3). These come together in the estate in *Top Boy* in a syndemic; the psychiatric morbidity and behavioural conditions are exacerbated and show synergy with the health inequalities caused by poverty, lack of green spaces, stress, structural violence and racial discrimination. We see how this leads to educational disadvantage through truancy in the cases of Ra’Nell and Ats, whose parents are suffering respectively from the mental health effects of depression and violence and in Ats mother’s case, forced unemployment and the threat of deportation. Such educational disadvantage makes individuals much more likely to become affiliated with gangs in a syndemic environment as more legal forms of employment become closed off to them in the setting of acute financial stress. Thus, the cycle of illegal activity, gang violence and mental ill health is perpetuated. The stories of individuals represented by Ra’Nell, Ats and Gem depict the potential long-lasting effects of the failure of mental health promotion as well as the timely identification of at-risk young persons within the context of mental illness preventative strategies. From a public health angle, mental disorder prevention is aimed at reducing the incidence (new cases), prevalence and recurrence of mental disorders as well as risk conditions for mental illness (17).

This creates both a challenge and also opportunity for public mental health in the context of social psychiatry. Black men are approximately half as likely as their White counterparts to use professional mental health services (35). Better understanding of why this is and working with these communities is crucial if we are to design appropriate interventions which are likely to be taken up, and break this cycle (36). Gang members currently will make a large contribution to mental health disability, service burden and the economic impact of criminal activity in syndemic areas (10). Given the proven links between gang membership and psychiatric service use, in particular traumatisation and fear of retaliatory violence (3), it is important to consider how psychiatry can promote disentanglement from gang affiliation by contributing to the multi-agency interventions required to reduce structural inequalities and circumstantial predispositions that exacerbate antisocial tendencies.

## Conclusion

*Top Boy* is a powerful lens through which the impacts of the syndemics of gang violence, racial trauma, urbanicity and poverty impact on black mental health can be interrogated. In line with the far-reaching social discourse to be found on social media platforms, it can help individuals relate themes in the show to their own lived experience. Indeed a likely factor for *Top Boy*’s success is its realistic and multi-faceted portrayal of the mental health difficulties faced by individuals in these environments, as can be seen from anecdotal reviews of the show by those who have been involved in gang violence or grown up in inner-city urban environments.

In a bi-directional way, the show can be a platform both for learning and education on the part of both the broader society and the groups which suffer these syndemics. Such learning falls under the umbrella of entertainment-education, and has been studied in relation to both health promotion and social change. In contrast to deliberately designed ‘edutainment’ shows which face the perhaps obvious pitfalls of feeling driven by underlying motive, research suggests that the effectiveness of these shows from a public health perspective are driven by their emotionality and effective illustration of both positive and negative behaviours (37). A show like ‘Top Boy’ may provide a particularly valuable and effective tool for addressing issues at hand given its naturalistic focus and evocative style. An understanding of how structural factors epidemiologically affect what psychiatric conditions individuals are likely to suffer, how they can be better reached by psychiatric services, and what cross-sectoral interventions can help improve the socioeconomic factors that lead to the behaviours/paths that individuals end up in will be vital for public mental health policy both now and in the future.

## Data availability statement

The original contributions presented in the study are included in the article/supplementary material. Further inquiries can be directed to the corresponding author.

## Author contributions

WQ: Conceptualization, Investigation, Writing – original draft, Writing – review & editing. AO: Investigation, Writing – original draft, Writing – review & editing. AS: Conceptualization, Investigation, Writing – original draft, Writing – review & editing.
